# The Elephant in the Mirror: Bridging the Brain's Explanatory Gap of Consciousness

**DOI:** 10.3389/fnsys.2016.00108

**Published:** 2017-01-06

**Authors:** Jasmine A. Berry, Alice C. Parker

**Affiliations:** ^1^Biomimetic Real-time Cortex Project, Computer Science Department, University of Southern CaliforniaLos Angeles, CA, USA; ^2^Biomimetic Real-time Cortex Project, Ming Hsieh Department of Electrical Engineering, University of Southern CaliforniaLos Angeles, CA, USA

**Keywords:** self-recognition, self-awareness, consciousness, machine intelligence, hard problem

## Introduction

The successes of the artificial retina and cochlea have lent encouragement to researchers in the general field of brain augmentation (Gantz et al., 2005; Dagnelie, 2012). However, in order for brain augmentation to progress beyond conventional sensory substitution to comprehensive augmentation of the human brain, we believe a better understanding of self-awareness and consciousness must be obtained, even if the “hard” problem of consciousness (Chalmers, 2007) remains elusive. Here we propose that forthcoming brain augmentation studies should insistently include investigations of its potential effects on self-awareness and consciousness. As a first step, it's imperative for comprehensive augmentation to include interfacing with the biological brain in a manner that either distinguishes self (biological brain) from other (augmentation circuitry), or incorporates both biological and electronic aspects into an integrated understanding of the meaning of self. This distinction poses not only psychological and physiological issues regarding the discrepancy of self and other, but raises ethical and philosophical issues when the brain augmentation is capable of introducing thoughts, emotions, memories and beliefs in such an integrated fashion that the wearer of such technology cannot distinguish his biological thoughts from thoughts introduced by the brain augmentation.

A consideration of self begins with the conventional mirror self-recognition test (MSR) (Gallop, 1970) that has been successfully executed with Eurasian magpies (Prior et al., 2008), bottlenose dolphins (Reiss and Marino, 2001), orca whales (Delfour and Marten, 2001), human infants typically between 18 and 24 months (Amsterdam, 1972; Rochat, 2003), and notably the Asian elephant (Plotnik et al., 2006). The only primate species reported to pass the Gallup Mirror Test, albeit controversially, were orangutans and chimpanzees (Suárez and Gallup, 1981). For years, MSR has been the designated litmus test for determining whether a species possesses self-awareness (SA), ultimately raising the question of whether the animal is then a conscious entity as a result of passing this test (De Veer and van den Bos, 1999). “Mirror self-recognition is an indicator of self-awareness,” proclaims Gallup et al. (2002). If indeed so, then the subsequent query to raise is whether self-awareness, the ability to differentiate oneself among others, is a precursor to or derivative of consciousness, and whether the mirror test is necessary and sufficient (Morin, 2011).

In light of brain research like the Blue Brain Project (Markram, 2006), BRAIN Initiative (Kandel et al., 2013), and development of neural prosthetics, the interest in consciousness is steadily growing. Here, we not only encourage the study of and suggest methods for addressing science's “*elephant in the room*,” which asserts consciousness is neither physical nor functional, but also place the *Elephas maximus* in our proverbial mirror to obtain a perspective toward forming a cohesive alliance between philosophical studies of consciousness and neural engineering's augmentative innovations. As MSR is purposed to grant the animal subject personal physical inspection from an objective viewpoint, resulting in self-cognizance, so shall we take the approach to examine our modern scientific methods in conceptual mirrors, to appraise our consciousness dilemma and propose an assertion for progression in augmentative technologies. Following here is a succinct primer of consciousness and SA. We also issue a proposition as to how brain augmentation can influence the arrival of machine consciousness. Overall, we state our opinion for (1) why SA must be systematically examined in conjunction with brain augmentation approaches and (2) how such a merger could become a tool for investigating consciousness.

### Ineffable consciousness

The first pitfall encountered with consciousness is the inability to derive a functional explanation for what it means to *experience*. Chalmers (2007) lists the “easy” problems of consciousness as “the ability to discriminate, categorize, and react to environmental stimuli; the integration of information by a cognitive system; the reportability of mental states; the ability of a system to access its own internal states; the focus of attention; the deliberate control of behavior; the difference between wakefulness and sleep.” These phenomena are relatively feasible to exploit and can be described in computational model terms and neural operation derivations. Chalmers then counteracts them with the “hard” problem of lacking competency to explain why and how we have phenomenal experiences when being entertained by a movie, exhibiting a sensation toward classical music, or having feelings when watching a sunset. Explaining how the brain processes visual and auditory signals is trivial in comparison to how those same signals translate to *qualia*, subjective phenomenal experiences.

### Explanatory gap dilemma

The term *explanatory gap*, coined by philosopher Joseph Levine (1983), notes our inability to connect physiological functions with psychological experience, thus creating the *gap*. Although Levine synonymizes consciousness with subjective feelings, the explanatory gap additionally alludes to reasoning, desires, memory, perception, beliefs, emotion, intentions, and human behavior/action. Correlating physical brain substrates to thoughts and feelings is the base of dispute between two parties: materialist reductionists and non-reductionists (Sawyer, 2002). Materialists' chief view, representative of most neuroengineers, on the matter involves the belief that “when the brain shuts off, the mind shuts off” and the brain is the sole causative driver for consciousness. However, non-reductionists (typically philosophers) embrace a holism approach of mandating that the brain's cortical components are insufficient in capturing consciousness, undertaking the possibility of supernatural properties. It's an inquiry of necessity and sufficiency. The brain may be necessary for mental functions, but is it sufficient? Earlier analytical inspections on conscious experience have implied that an exclusive reductive justification is not satisfactory in delineating its emergence (Churchland, 1988; Kim, 2005; Clayton, 2006; Feinberg, 2012). A novel approach is needed to explain such experience. Our explanatory gap needs an explanatory bridge.

## Unraveling self-awareness toward augmentation

**Figure 1:**
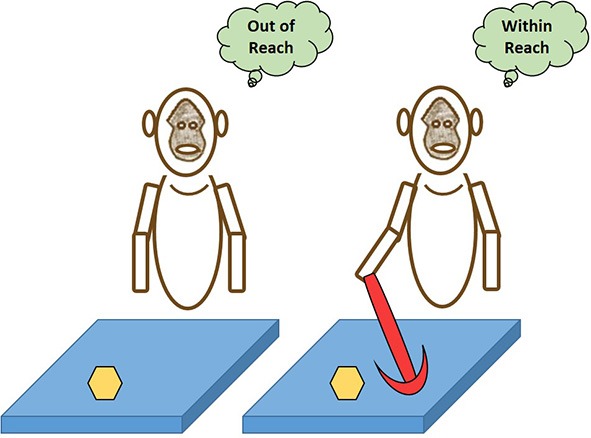
**Extension of self-representation**. Here are two depictions of macaque monkeys that exhibit a case of the body making use of tools as an extension of the “self.” If given a task to retrieve an object (yellow hexagonal shape) that is outside the peripersonal space and the immediate reach of an extended limb (left macaque), the body relies on its physical limitations to define the “self” and its aptitude for success of the task. However, when an apparatus is introduced (right macaque) that can help achieve the task's goal, the brain's neural correlates are able to augment themselves to psychophysically merge tools that were formerly considered to be of “other” classification into the “self” body schematic and permit optimal behavioral actions to take place (Hihara et al., 2006; Carlson et al., 2010). The paradigm for “self” is malleable to accept the dynamic interplay necessary to achieve an aim for biological function that was once previously unattainable. As tool-use changes the brain's representations of the body and alters proprioception, we subsequently believe it parallels how enriched brain augmentation can alter an individual's self-awareness and consciousness.

Although many facets of consciousness are difficult to investigate, the development of objective tests for SA could be utilized for brain augmented technologies. With SA comes the sense of *agency*. Agency imparts a sense of who is the owner of an action/trait, the self, and who represents any entities excluding self, the other(s). Self-other dichotomy processing in the brain is essential to consciousness due to the necessary implications the embodiment of “self” must have to form body ownership. Once an agent gains the ability to discern when its own body is the source of sensory perceptions, it will form body awareness that entails proprioceptive information. We can look to working experiments that attempt to showcase how the brain augments the “self” when necessary to complete a task (Figure 1). Perceptual parametric information builds a premeditated awareness of (1) body part locations and (2) the manipulation of those same parts in space. Body awareness was demonstrated by a machine via Gold and Scassellati (2007) who built a robot named Nico that successfully distinguished its own “self” from “other.” Nico observably achieved self-recognition by completing mirror-aided tasks expending inverse kinematics. Nevertheless, it's believed Nico lacked consciousness.

Before the sense of agency becomes fully refined through experiences over time, there must be a repertoire built for perceptions and actions. Whether, action and perception are interdependent or each fundamentally isolated has been the focus of another ongoing debate. It's not yet concretely understood how the representation of self is formed during the initial stages of life. Either an agent first uses perception to motivate their actions in the world or it first directs their actions to help drive perception of the sensory world or both occur simultaneously. In either method bodily awareness is eventually acquired which contributes to defining subjective cognitive attributes. Two opposing views attempt to solve this problem: the action-oriented theory of visual perception, which suggests that perception results from sensorimotor dynamics in an *acting* observer (Gibson, 1966; Noë, 2004; Mandik, 2005), and the dual-visual systems hypothesis, which advocates independent streams of perception and action (Schneider, 1969; Goodale and Milner, 1992; Jacob and Jeannerod, 2003; Milner and Goodale, 2006). Self-awareness uses expectation of impending perceptions and actions to gauge the assimilation of inner experience and external reality. Building a self-aware framework in augmentative technologies requires integration of an *expectancy intuition*, which is the capability to critique on the basis of differences between reality and internal experience. This is our tactic for creating systems with faculties for using perception and action to make predictions of self-sensory states, become self-adaptable to new environmental stimuli, and set objectives for self-improvement.

Crucial for understanding agency is determining how the embodied senses fuse to form self-referential experience (Fingelkurts et al., 2016a,b). It's our opinion that future advances of brain augmentation hinges on the application of such knowledge. Once we bridge this gap of the unknown we'll be challenged to use computational intelligence to create consciousness artificially and to integrate synthetic qualia with that produced in the brain. Presently, artificial devices can create various aspects of consciousness. Artificial perception is made available via cochlear, retinal, and tactile implants. But they simply work alone as replacements for sensory organs with consciousness and SA arriving later in the brain's neural processing. Applications for augmenting consciousness would contribute to studies relating to emotions, attention, supplementing memory capacity, personality alteration, experience enrichment, sensory perception enhancement, and hypernormal brain plasticity for self-repair.

## Proposed transition to machine consciousness

The marvel of human intelligence is its ability to eclipse physical limitations and overcome our biological constraints to form an ever-evolving existence (Jerison, 1973). One primary goal for reverse-engineering the human brain is to recreate the same functional mechanisms that underlie human consciousness in our software infrastructures, neurorobotic agents, and computational systems. However, prosthetic memory, sensory implants, neurofeedback (EEG Biofeedback) and brain computer interfaces (BCIs) are all working examples of fusing such “intelligent” systems with the brain, leading to conceivable prospects for consciousness-altering devices. Although BCIs commonly target disability treatments and brain function recovery from lesion, the amalgamation of computational devices with the cortical brain itself (Fingelkurts et al., 2012) may even prompt increasing developments of an operational “exobrain” (Bonaci et al., 2014) for the purposes of better understanding how our brain works. For example, in a scenario where a split-brain condition is present within a subject, we now have the option to look toward interfacing artificial exobrains with the cerebrum; such an interface can either serve as a replacement for neurological issues or supplement features the brain does not naturally comprise. If these exobrains have a modicum of manipulability, then we can explore the plausibility of mind transfer from device to organ and vice versa; thus, providing speculation for a conscious machine that can affect how we can perceive, act, express emotion, feel, and adapt. This poses ethical concerns as it opens the door for alterations of an individual's SA when augmentation is capable of modifying reasoning skills and subjective judgment. Successful augmentation of the sort might render the individual powerless in discriminating actual characteristics and thoughts from those that are mock and introduced artificially outside the cortex. Combining the precision and information processing speed of a computer with the intrinsic non-computational attributes of a human may provoke discoveries of the mind (e.g., consciousness) that we as humans are currently incapable of resolving. We suggest efforts made toward an augmentative interface between brain and machine that prompts the human mind to think beyond its unknown limits for the construction of our explanatory bridge.

## Challenges moving forward

Many people view an in-depth exploration into consciousness and its emergence as a gamble, considering decades already spent on the matter with a void of consensus (Dennett, 1991; Jibu and Yasue, 1995; Hameroff and Penrose, 1996; Stapp, 2001; Crick and Koch, 2003; Tononi, 2008; De Sousa, 2013; Seager, 2016). Before we attempt to create another hypothesis, our approach needs to change; it's our suggestion to further refine the constructs and emergence of SA and to use brain augmentation as an instrument for inspection. We need to define an objective test for determining whether an entity is a sentient being. This test in addition to advances in neural engineering provide optimism that disputes within the consciousness field can be resolved. Augmentation has a promising future as an enhancement to our brains and will hopefully influence our centuries-old methods of thinking about consciousness toward an answer for science's greatest mystery.

## Author contributions

JB: This author was responsible for providing the overall opinion of the topic and crafting the outline of the article. JB contributed most of the text and references supplied in the document. JB also primarily conducted the necessary research to develop this article. JB supplied the latter sections of the text with original ideas to add to the discussion of the Brain Augmentation research topic. AP: This author assisted in crafting the thesis of the article and provided additional topic of discussion to implement within the text. AP provided introductory material for the article and also made revisions to each section to ensure a coherent message about our opinion was made. AP also restructured the format of the article to enhance comprehension to an audience with varying backgrounds.

### Conflict of interest statement

The authors declare that the research was conducted in the absence of any commercial or financial relationships that could be construed as a potential conflict of interest.
